# Protocol to determine *in vitro* lipid-protein interactions using liposome flotation assay

**DOI:** 10.1016/j.xpro.2026.104543

**Published:** 2026-05-07

**Authors:** Karan Khadayat, Morgan House, Vasudeva Tati, Amit S. Joshi

**Affiliations:** 1 Department of Biochemistry & Cellular and Molecular Biology, University of Tennessee, Knoxville, TN 37916, USA

**Keywords:** Cell culture, Cell isolation, Cell Membrane, Cell-based Assays, Cell separation/fractionation, Flow Cytometry

## Abstract

Lipid-protein interactions are crucial for cellular metabolism, transport, and signaling. Here, we present a detailed protocol to determine lipid-protein interactions using purified proteins, commercially available lipids, and a gradient centrifugation-based technique. We show how to make liposomes, test liposome size, form sucrose gradients with liposomes and purified protein, and collect and analyze the fractions after centrifugation. This protocol can determine lipid-protein interactions using a variety of liposome compositions for different proteins.

For complete details on the use and execution of this protocol, please refer to House et al.^1^

## Highlights

• Protocol to determine lipid-protein interactions
• Steps for preparing unilamellar liposomes
• Instructions for fraction collection and analysis using immunoblotting

## Abstract (editor highlights)

Publisher’s note: Undertaking any experimental protocol requires adherence to local institutional guidelines for laboratory safety and ethics.

## Graphical abstract

**Figure:**
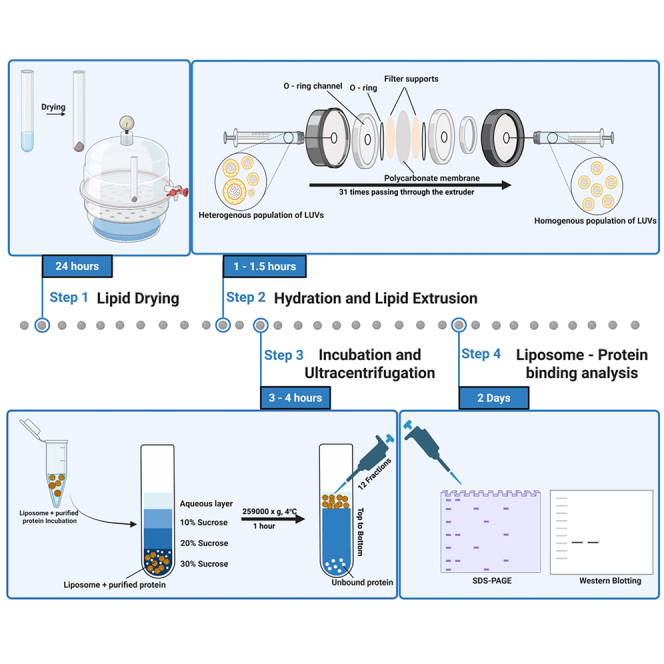

## Before you begin

### Innovation

Several biochemical assays are available to study lipid-protein interactions, including surface plasmon resonance (SPR), isothermal titration calorimetry, tryptophan fluorescence, lipid overlay, and co-sedimentation assay.^2^^,^^3^ Among these techniques, pitfalls include non-specific binding, a large amount of starting material needed, and artifacts due to oligomerization of the protein of interest.^3^^,^^4^^,^^5^ Here, we present a liposome flotation technique to assess lipid-protein interactions. This technique is not sensitive to protein oligomerization and aggregation, and also mimics the curvature of cellular membranes.^2^^,^^3^^,^^6^^,^^7^ Additionally, less starting material is needed for this assay as protein concentrations in the 1–4 μM range are sufficient. Thus, this strategy can be used for any purified protein to study its interaction with lipids.

1. Purify protein of interest using standard purification techniques (e.g., affinity chromatography, size exclusion chromatography).

***Note:*** We have used hexahistidine-(6×His) tagged proteins, but any tag (e.g., FLAG, HA) on your protein of interest should be sufficient to use in this assay.

2. Ensure that lipids are fresh and not past their expiration date. Using old lipids can affect liposome preparation.
3. Freshly prepare the liposome buffer before use.

### Lipid drying

**Timing: 25 h**

4. Lipid Stock Preparation: Obtain commercially available lipids from Avanti Polar Lipids and dissolve each lipid in chloroform according to the manufacturer’s instructions to prepare individual stock solutions.

***Note:*** We have used phosphatidylcholine (PC), phosphatidylethanolamine (PE), and phosphatidic acid (PA) in our liposome preparations. PC, PE, and PA are dissolved in chloroform. Other lipids could be used in liposome preparations and should be dissolved according to the manufacturer’s instructions.

5. In a clean long glass tube, mix lipids to a final total concentration of 2mM in the following molar ratio: PC:PE: PA = 70:20:10 mol%. (Table 1).

**Table 1:** Liposome composition for PC:PE:PA- 70:20:10 mol%

Lipid type (mol %)	Stock concentration (mM)	Working concentration (mM)	Volume (μl)
PC (70 mol %)	13.44	1.4, 1.5 or 1.6	33.01
PE (20 mol %)	12.72	0.4	8.92
PA (10 mol %)	13.83	0.2	4.33

***Note:*** The lipid composition could be determined based on the membrane of interest.

**CRITICAL:** Use a glass syringe to dispense lipids into the glass tube if lipids are in chloroform.

6. Evaporate the chloroform under a gentle stream of argon gas to form a thin, uniform lipid film on the inner wall of the glass tube.

**CRITICAL:** Ensure no clumps remain at this stage and that lipids are completely dried.

7. Place the tubes in a desiccator under vacuum for 24 h at 20°C to ensure complete drying of the lipid film.

**Pause point:** Lipids dried under vacuum for 24 h can be stored in glass tubes at −20°C for future use. Dried lipids should remain stable for 6 months at −20°C.

### Hydration and freeze-thaw cycles

**Timing: 1 h**

8. After lipids have been under vacuum for 24 h, rehydrate the lipid film in liposome buffer.

***Note:*** The volume of liposome buffer used to reconstitute lipids will vary according to number of reactions.

9. Resuspend vigorously for 5–10 min using a mini vortex mixer to dissolve the lipids.

**CRITICAL:** Ensure that no lipid film remains on the side of the tube.

10. Perform 10 freeze-thaw cycles using liquid nitrogen and a 42°C water bath. Freeze the sample completely in liquid nitrogen and then thaw it completely in a water bath.

**CRITICAL:** Complete freezing and thawing are critical for optimal lipid dispersion and extruder performance.

### Lipid extrusion

**Timing: 10–20 min per liposome type**

11. While freeze-thawing lipids, assemble the mini extruder according to the manufacturer’s instructions.
  a. Place membrane supports on the flat surface with O-rings facing up.
  b. Soak the filter in ddH_2_O or liposome buffer and place it inside the O-ring diameter.
  c. Place the polycarbonate membrane (100 nm) over the filter support.

***Note:*** See the graphical abstract for the assembly diagram.

12. Carefully place the second internal membrane support into the casing with O-rings facing down, without twisting on contact with the membrane.
  a. Once inserted, tighten the retainer nut by hand to the threaded end of the extruder outer casing until finger tight.
  b. Place the extruder stand onto a hot plate and insert the thermometer into the well provided in the heating block. Leave until it reaches 45°C.
13. Load liposome buffer into one extruder syringe and insert both syringes into the extruder assembly. Ensure the empty syringe is set to zero, and swing-arm clips that hold syringes in place are tight. Press the syringe that contains the liposome buffer to pass the buffer through the extruder assembly into the empty syringe on the other side.
  a. Perform 10 – 12 passes through the extruder to ensure there is no leakage. You should end up with roughly the same volume as loaded if the extruder is assembled properly. See troubleshooting, problem 1 if leaking occurs.
14. Load the lipid sample into one of the extruder syringes and place it into one end of the extruder assembly. Place the empty syringe, set to zero, on the other side of the extruder and ensure swing-arm clips are tight.
15. Leave the extruder for 5–10 min in the extruder stand until the lipid suspension equilibrates with the heating block.
16. Press the syringe containing the lipid solution until the solution is completely transferred to the other empty syringe. Gently push the now full syringe plunger to transfer the solution back to the original syringe.
  a. Repeat this step for 31 passes through the membrane to ensure uniform vesicle size. Always perform an odd number of passes to collect lipids from the opposite loading side to avoid the larger particles or foreign material.
17. Remove the filled syringe from the extruder and transfer liposomes to a clean 1.5mL Eppendorf tube.

***Note:*** A new filter support and polycarbonate membrane should be used for each liposome preparation. Extrusion assembly should be washed before each liposome preparation.

**CRITICAL:** The syringe should be removed straight, parallel to the extruder holder; removing at an angle can damage or crack the syringe.

**Pause point:** Extruded liposomes can be stored at 4^o^C for 4–5 days. However, we recommend that you use freshly made liposomes for ultracentrifugation.

### Size and homogeneity analysis of liposomes by dynamic light scattering

**Timing: 1–2 h**

18. Determine the size distribution and homogeneity of extruded liposomes using Dynamic Light Scattering (DLS), a reliable technique for characterizing nanoparticle size in solution. Transfer 100 μL of the liposome suspension into a cuvette compatible with the DLS instrument.
  a. Use a liposome buffer without liposomes as the blank control to account for background scattering.
19. Use a DLS instrument and the DYNAMICS program to measure liposome diameter and polydispersity.

**Figure 1:**
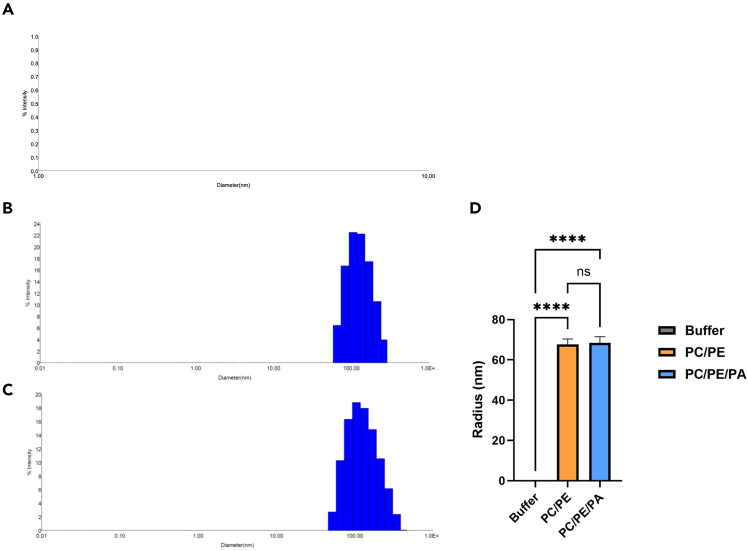
Preparation of unilamellar liposomes by extrusion of large multilamellar vesicles Dynamic light scattering data for liposomes obtained by extrusion of large multilamellar vesicles (A) Buffer only, (B) PC/PE liposomes, (C) PC/PE/PA liposomes, (D) Quantification of liposomes. Data represented as mean ± SEM for n ≥ 3 experiments, ∗∗∗∗*p*, 0.0001, statistical analysis was performed using the one-way ANOVA with multiple comparisons.

***Note:*** No liposome were detected in buffer only (Figures 1A and 1D). Our measurements confirmed that the average size of PC/PE liposomes was 67.697 nm (Figures 1B and 1D), while PC/PE/PA liposomes measured 68.453 nm on average (Figures 1C and 1D), indicating that the extrusion procedure produced highly homogeneous liposome preparations.

## Key resources table

**Table:** 

REAGENT or RESOURCE	SOURCE	IDENTIFIER
**Antibodies**		

6×-His Tag Monoclonal Antibody (HIS.H8), 1:2000	Invitrogen	Cat.#MA1-21315; RRID: AB_557403
IRDye® 680RD Donkey anti-Mouse, 1:5000	LI-COR Biotech LLC	Cat.#926-68072

**Chemicals, peptides, and recombinant proteins**		

18:1 (Δ9-Cis) PC (DOPC) 1,2-dioleoyl-sn-glycero-3-phosphocholine	Avanti Research	Cat.#850375 P-25mg
18:1 (Δ9-Cis) PE (DOPE) 1,2-dioleoyl-sn-glycero-3-phosphoethanolamine	Avanti Research	Cat.#850725 P-25mg
18:1 PA, contains: chloroform 1,2-dioleoyl-sn-glycero-3-phosphate (sodium salt)	Avanti Research	Cat.#840875C-25mg

**Software and algorithms**		

DYNAMICS 7.1.6.5	Wyatt Technology	2–4

**Other**		

Extruder Set without Holder/Heating Block	Avanti Research	Cat.#610023-1Ea
Polycarbonate Centrifuge Tubes (11 × 34mm)	Beckman Coulter	Cat.#343778
Fisherbrand™ Round Bottom Disposable Borosilicate Glass Tubes with Plain End	Fisher Scientific	Cat.#14-961-27
Vacuum desiccator	N/A	N/A
Fisherbrand™ Variable Speed Mini Vortex Mixer	Fisher Scientific	Cat.#14-955-163
Heat block	N/A	N/A
Wyatt Industries WDPN-04 DynaPro NanoStar Nano Particle Detector	Wyatt Technology	N/A
TLS-55 Swinging-Bucket Rotor Package	Beckman Coulter	Cat.#346134
Optima™ Max-XP Tabletop Ultracentrifuge	Beckman Coulter	Cat.#393315
LI-COR Odyssey CLx Imager	LICORbio™	N/A

## Materials and equipment

• Liposome buffer: 50 mM NaCl and 25 mM Tris-HCl, pH 7.5 in ddH_2_O.

## Step-by-step method details

### Liposome-protein incubation and sucrose density gradient preparation

**Timing: 1– 2 h**

The following steps detail how to make the liposome and protein solution and how to make a sucrose density gradient for centrifugation.

1. Add purified protein at a final concentration of 4 μM and extruded liposomes at a final concentration of 2 mM in a total volume of 60 μL to an Eppendorf tube. Use liposome buffer to bring the total volume to 60 μL.
  a. As a negative control, mix purified protein at a final concentration of 4 μM in 60 μL of liposome buffer, without liposomes.
2. Incubate the mixture at 20°C for 1 hour while gently rocking.

***Note:*** To address non-specific interaction between protein and lipids include a negative control. Use a known protein that does not bind lipids.

3. Prepare 60%, 20%, and 10% (w/v) sucrose solutions in liposome buffer during the incubation period.
4. Transfer the liposome-protein mixture (60 μL) to the bottom of a polycarbonate centrifuge tube.
  a. Add 60 μL of the 60% (w/v) sucrose solution to the liposome and protein mixture, resulting in a 30% sucrose-liposome-protein mixture, and
  b. Mix well by gentle pipetting.
5. Carefully pipette 240 μL of the 20% sucrose solution on top of the 30% sucrose-liposome-protein mixture. **Do not mix.**
6. Carefully pipette 240 μL of the 10% sucrose solution on top of the 20% sucrose solution. **Do not mix.**
7. Carefully pipette 120 μL of liposome buffer on top of the 10% sucrose solution. **Do not mix.**

**CRITICAL:** Making the sucrose gradient is a crucial step**.** Carefully pipette the solutions on top of the other by touching the side wall of the polycarbonate tube with the pipette tip and slowly releasing the solution down the side of the wall. Ensure you are not disturbing the layers. If a gradient is formed properly, the different layers should be visible.

***Alternatives:*** Instead of sucrose, Nycodenz can be used to make the gradient.

### Centrifuging the gradients and collecting the fractions

**Timing: 1.5–2 h**

The following steps detail how to centrifuge the gradients and how to collect the gradient fractions.

8. Carefully transfer the polycarbonate tubes containing the sucrose gradients to a TLS-55 swinging-bucket rotor in a Beckman Coulter ultracentrifuge without disturbing the gradients. Centrifuge samples at 259,000 *× g* for one hour at 4°C.

***Optional:*** Careful pipetting should result in each polycarbonate tube having an equal amount of volume. However, to ensure that each tube weighs approximately the same, weigh the tubes before adding them to the centrifuge and balance tubes in the rotor accordingly. Balanced tubes should not exceed a 0.04g difference since centrifugation speeds are high. If needed, add liposome buffer to the top gradient to balance the tubes.

9. Carefully remove the polycarbonate tubes from the rotor without disturbing the gradients.
  a. Pipette 12 fractions of 60 μL each from the tubes, starting at the top of the gradient.
  b. Place each fraction in labeled Eppendorf tubes 1–12, with the first collected fraction from the top being tube #1.

***Note:*** To determine liposome migration in the gradient, we recommend using fluorescently tagged lipids and lipid only control.

**CRITICAL:** Removing the fractions from the gradient is a crucial step. Be careful not to disturb the gradients. Place the pipette tip on the side of the polycarbonate tube wall, just touching the top of the liquid layer. Slowly pull up the fractions into the tip and continue until you have reached the 12^th^ fraction.

10. Store collected fractions at −20°C until the time of immunoblot analysis.

**Pause point:** Collected fractions can be stored until immunoblotting.

### Immunoblotting of fractions and analysis of results

**Timing: 2 days**

The following steps show how to analyze the gradient fractions using immunoblotting and how to interpret the results.

11. Mix each fraction with an appropriate amount of SDS sample buffer and load fractions sequentially in an SDS-PAGE gel.
12. Perform the standard immunoblotting procedure using primary antibodies that are reactive with your protein of interest. Apply secondary antibody and scan the blots using a machine compatible with detecting the secondary antibody.

***Note:*** We have used primary antibodies that are reactive with our protein tag (6×-His); however, primary antibodies against the protein itself should work in this assay. We have also used IR secondary antibodies compatible with the LI-COR systems; however, other secondary antibody types and scanning systems should be compatible with this assay.

**Figure 2:**
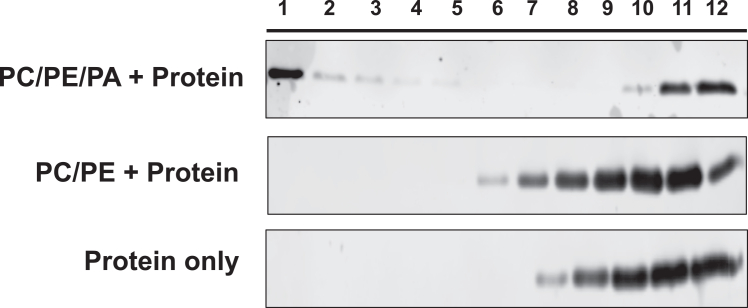
Analyzing lipid-protein interaction by immunoblot Twelve fractions of 60μl each were subjected to western blot using anti-6×HIS monoclonal antibody to check for the presence of protein of interest tagged with 6×HIS.

13. After developing the blots, analyze the images to determine lipid-protein interactions. Lipid protein interactions are confirmed by the presence of protein bands in the top fractions of the gradient. A negative control blot should show protein bands only in the bottom fractions of the gradient (Figure 2). See troubleshooting, problems 2, 3, and 4 for confounding immunoblot results.

## Expected outcomes

This protocol enables robust and reproducible detection of lipid-protein interactions using a liposome flotation assay. Upon successful execution, liposomes with bound recombinant protein will migrate to the top fractions of the gradient after ultracentrifugation, while unbound proteins remain in the lower fractions. Western blot analysis should reveal clear protein bands in the upper fractions only when the protein interacts with liposomes, confirming specific lipid-protein binding. Control experiments lacking liposomes are expected to show little or no signal in the flotation (top) fractions. The size analysis by dynamic light scattering (DLS) should indicate formation of unilamellar vesicles with low polydispersity, supporting homogeneity of the liposome preparations and reliability of subsequent binding results. This protocol can be adapted for different lipid compositions or protein constructs, making it suitable for broad applications in membrane protein and lipid-interaction studies.

## Limitations

Several experimental limitations must be considered with this assay. The requirement of an ultracentrifuge with compatible rotors may limit accessibility for some laboratories, as these instruments are costly and specialized. Improper gradient layering can introduce variability or artifacts. The assay detects co-migration of protein with liposomes but does not inform on the binding kinetics, affinity, or specific binding sites. Proteins that interact weakly or transiently with membranes may dissociate during ultracentrifugation, leading to underestimation of binding. Additionally, this method is less suitable for studying dynamic or reversible lipid-protein interactions that occur on rapid timescales. Finally, for proteins or lipids sensitive to buffer conditions, temperature, or freeze-thaw cycles, additional optimization may be required to preserve function and reproducibility.

## Troubleshooting

### Problem 1

Extrusion leakage (before you begin Step 13).

### Potential solution

The filter supports and/or polycarbonate membrane are not properly inserted between the O-rings. Disassemble the extruder and remove filter supports and polycarbonate membrane. Dry the assembly. Soak fresh filter supports in ddH_2_O or liposome buffer and dab off excess liquid. The filter supports should fit perfectly inside the black circle of the O-ring. Ensure that the filter supports are flat against the O-rings and not wrinkled. Place one O-ring inside the outer metal casing. Carefully place the polycarbonate membrane inside the metal casing on top of the filter support on the O-ring. The polycarbonate membrane should not be soaked in liquid before placing it in the extruder. Ensure that the polycarbonate membrane is flat against the O-ring with the filter support and minimize the amount of wrinkles in the membrane. Place a fresh, soaked filter support on the other O-ring, and be careful not to twist the O-ring when inserting it into the metal casing. This could wrinkle the polycarbonate membrane and result in a loose fit.

### Problem 2

Protein bands were detected in all gradient fractions (Step 13).

### Potential solution

Protein detected in all fractions can be a result of an error in the preparation of the sucrose gradient or a result of a disturbed sucrose gradient. When making the gradient, ensure the layers are not disturbed. Place your pipette tip just above the previous gradient and pipette gently down the side wall of the polycarbonate tube. Be careful when moving the samples and keep them steady when placing them inside the centrifuge rotor.

Additionally, protein could be in all fractions due to mistakes in collecting the gradient fractions. Gently collect the fractions in the opposite way of making the gradient: place your pipette tip at the very top of the liquid layer next to the tube wall and slowly pull up each fraction. Make sure that your pipette tip is not placed too far down into the liquid, resulting in inaccurate fractions.

Using a high concentration of protein in the assay could lead to detection in all fractions if your protein of interest does indeed bind to the lipids you are testing. Ideally, the intensity of protein in the middle fractions should be undetectable or lower than the upper fractions. Optimize protein concentrations to reduce the presence of protein in all fractions.

### Problem 3

No protein detected in immunoblot (Step 13).

### Potential solution

It is possible that the suggested pH (7.5) or buffer is not suitable for your protein of interest. Test different pH levels or buffers such as HEPES with NaCl to find which buffer is suitable for your protein. Additionally, adding 0.5–1 mM of reducing agents such as dithiothreitol or β-mercaptoethanol can prevent aggregation or oxidation of proteins with cysteine residues.

### Problem 4

No protein detected in top gradient fractions of a positive control sample (Step 13).

### Potential solution

Optimize incubation step (Step 2). Ensure the liposome-protein mixture is gently rocking or rotating. Longer incubations at 20°C could yield more robust binding. Additionally, incubations at 4°C with gentle rocking or rotation could be more optimal for the binding of your protein of interest. If incubating for 16 h, opt for incubation at 4°C.

## Resource availability

### Lead contact

Further information and requests for resources and reagents are available from the corresponding author, Amit S. Joshi (ajoshi18@utk.edu), upon reasonable request.

### Technical contact

Further information and requests for resources and reagents are available from the corresponding author, Amit S. Joshi (ajoshi18@utk.edu), upon reasonable request.

### Materials availability

This study did not generate new unique reagents.

### Data and code availability

This study did not generate/analyze datasets or code.

## Acknowledgments

Research reported in this publication was supported by 10.13039/100000002NIH award R35 GM147189 and startup funds from 10.13039/100007135University of Tennessee at Knoxville to A.S.J. M.H. was supported by 10.13039/100000002NIH T32 award GM142621.

## Author contributions

A.S.J. and K.K. developed the protocol. K.K., M.H., and V.T. conducted the experiments and data analysis. M.H., K.K., V.T., and A.S.J. wrote the protocol.

## Declaration of interests

The authors declare no competing interests.
